# The superior growth of *Kluyveromyces marxianus* at very low potassium concentrations is enabled by the high-affinity potassium transporter Hak1

**DOI:** 10.1093/femsyr/foae031

**Published:** 2024-10-03

**Authors:** Klara Papouskova, Joel Akinola, Francisco J Ruiz-Castilla, John P Morrissey, Jose Ramos, Hana Sychrova

**Affiliations:** Laboratory of Membrane Transport, Institute of Physiology, Czech Academy of Sciences, 142 00 Prague 4, Czechia; School of Microbiology, SUSFERM Fermentation Science Centre, University College Cork, Cork T12 K8AF, Ireland; Department of Agricultural Chemistry, Edaphology and Microbiology, University of Córdoba, E-14071 Córdoba, Spain; School of Microbiology, SUSFERM Fermentation Science Centre, University College Cork, Cork T12 K8AF, Ireland; Department of Agricultural Chemistry, Edaphology and Microbiology, University of Córdoba, E-14071 Córdoba, Spain; Laboratory of Membrane Transport, Institute of Physiology, Czech Academy of Sciences, 142 00 Prague 4, Czechia

**Keywords:** *Kluyveromyces marxianu*, potassium, transporter, uniporter, K^+^–H^+^ symporter, affinity

## Abstract

The non-conventional yeast *Kluyveromyces marxianus* has recently emerged as a promising candidate for many food, environment, and biotechnology applications. This yeast is thermotolerant and has robust growth under many adverse conditions. Here, we show that its ability to grow under potassium-limiting conditions is much better than that of *Saccharomyces cerevisiae*, suggesting a very efficient and high-affinity potassium uptake system(s) in this species. The *K. marxianus* genome contains two genes for putative potassium transporters: *KmHAK1* and *KmTRK1*. To characterize the products of the two genes, we constructed single and double knock-out mutants in *K. marxianus* and also expressed both genes in an *S. cerevisiae* mutant, that lacks potassium importers. Our results in *K. marxianus* and *S. cerevisiae* revealed that both genes encode efficient high-affinity potassium transporters, contributing to potassium homeostasis and maintaining plasma-membrane potential and cytosolic pH. In *K. marxianus*, the presence of *HAK1* supports growth at low K^+^ much better than that of *TRK1*, probably because the substrate affinity of *Km*Hak1 is about 10-fold higher than that of *Km*Trk1, and its expression is induced ~80-fold upon potassium starvation. *Km*Hak1 is crucial for salt stress survival in both *K. marxianus* and *S. cerevisiae*. In co-expression experiments with *Sc*Trk1 and *Sc*Trk2, its robustness contributes to an increased tolerance of *S. cerevisiae* cells to sodium and lithium salt stress.

## Teaser

Yeast *Kluyveromyces marxianus* grows at low KCl concentrations thanks to a powerful potassium transporter.

## Introduction

Like all organisms, yeast cells need to accumulate relatively high potassium concentrations, effectively regulate their fluxes across the plasma membrane in response to changing external conditions, and maintain their appropriate and different concentrations in particular organelles. Potassium fulfils many physiological roles, such as compensating negative charges in many macromolecules (e.g. DNA and RNA), regulating cell volume and intracellular pH, maintaining stable potential across cell membranes, and participating in protein synthesis or enzyme activation (Arino et al. 2010, Arino et al. 2019).

In general, yeast cells can grow at a broad range of external K^+^ concentrations (~10 µM–2.5 M), and they use several types of transporters to maintain appropriate potassium homeostasis and optimum concentration inside the cells (~200–300 mM). To accumulate potassium, most of the yeast species simultaneously employ Trk uniporters and Hak K^+^–H^+^ symporters and a very small number of yeast species also have a K^+^-uptake ATPase (Acu). To eliminate surplus potassium, yeasts use highly conserved monovalent-cation/H^+^ Nha antiporters and monovalent-cation efflux Ena ATPases (Ramos et al. 2011).

The Trk-type transporters have been studied much more than the Hak ones, as the model yeasts *Saccharomyces cerevisiae* and *Schizosaccharomyces pombe* have no Hak transporters. *Saccharomyces cerevisiae* only has two Trk transporters. Their absence in cells results in an inability to grow under low potassium conditions and also in changes in basic physiological parameters such as the level of plasma-membrane potential and intracellular pH (Navarette et al. 2010). Potassium uptake via Trk systems has been shown to be essential for stress survival, e.g. the desiccation of cells (Borovikova et al. 2014), or toxic effects of ethanol (Lam et al. 2014), organic acids (Xu et al. 2019), and a high concentration of ammonium (Hess et al. 2006, Reisser et al. 2013), pointing to the irreplaceable role of efficient Trk-mediated potassium uptake under many (adverse) environmental or biotechnology conditions. Though the Trk transporters of different yeast species share a very high level of sequence similarity and regulation of activity (switching from a low- to a high-affinity mode if potassium is present in scarce amounts in the environment) (Ramos et al. 2011), their activity and contribution to cell growth and stress tolerance differ significantly. Some of them, e.g. Trk1 of *Yarrowia lipolytica*, can improve the growth properties and stress tolerance of *S. cerevisiae* cells co-expressing *YlTRK1* and its own two Trk transporters (Papouskova et al. 2023).

Hak1 transporters have only been characterized from a few yeast species, e.g. osmotolerant *Debaryomyces hansenii* or pathogenic *Candida albicans*, and usually only upon their expression in *S. cerevisiae* lacking its own Trk transporters (e.g. (Prista et al. 2007, Elicharova et al. 2016, Ruiz-Castilla et al. 2021a)). In *S. cerevisiae*, they are usually able to compensate for the absence of Trks in terms of growth at low potassium, but except for *C. albicans* Hak1 (Ruiz-Castilla et al. 2021b, 2022), we do not know very much about whether and how Hak systems are involved in the maintenance of basic physiological parameters and the stress tolerance of the heterologous host.

*Kluyveromyces marxianus* (formerly also *Candida kefyr*) is a close relative of well-studied *K. lactis*. It is known mainly through isolates from foods and beverages, especially dairy products, but also in decaying plant tissue and associated insects (Kurtzman et al. 2010). Its high-speed growth, natural resilience against everyday industrial stresses, and recent advances in the development of molecular biology tools to perform strain engineering make *K. marxianus* a promising microbial cell factory candidate for the sustainable production of biochemicals (Morrissey et al. 2015). Although many aspects of its performance have been described and its complete genome has been sequenced (~10 Mb in eight chromosomes), more information is needed about *K. marxianus*’ potassium requirements and the transporters involved in maintaining its potassium homeostasis.

In the work reported here, we constructed single and double *K. marxianus* mutants lacking one or both potassium uptake systems, characterized their growth properties, stress tolerance, cation-uptake kinetics, *TRK1* and *HAK1* expression, as well as basic physiological parameters of the wild type and mutants. In parallel, we expressed both genes in an *S. cerevisiae* strain (BYT12) lacking its potassium importers (*trk1Δ trk2Δ*) and studied the properties of the engineered strains to compare the performance of both transporters. Finally, we expressed *KmHAK1* in several *S. cerevisiae* strains and checked whether this foreign gene could improve the properties of strains possessing functional *TRK1* and *TRK2*.

## Material and methods

### Yeast strains and growth media

The yeast strains used in this study are listed in Table S1. Cells were routinely cultivated at 30°C in rich YPD (1% yeast extract, 2% peptone, 2% glucose, Formedium), in minimum YNB (0,67% YNB, Difco, containing ~15 mM K^+^, 2% glucose) and low-potassium YNB-F (0.17%, Yeast nitrogen base w/o nitrogen source, Formedium, containing only 15 µM K^+^ (Navarette et al. 2010), 2% glucose) media as indicated in the text. YNB and YNB-F media were also supplemented with the oMM mixture of amino acids [from a 50× concentrated stock solution; (Hanscho et al. 2012)] for BY4741-derived strains or appropriate auxotrophic supplements (15 µg/ml); 2% agar was used for plates.

For YNB-F, 0.4% (NH_4_)_2_SO_4_ was generally used as a nitrogen source. Proline (0.1%) was used instead of ammonium sulfate in one indicated experiment. The pH of YNB-F was adjusted to 5.8, either with NH_4_OH (NH_4_^+^ used as the nitrogen source) or triethanolamine (medium supplemented with proline). To study the effect of various extracellular pH levels, YNB-F was buffered with 20 mM MES (pH 5.8) or 20 mM MOPS (pH 7.0), and the pH level was adjusted with NH_4_OH. Tartaric acid was used to adjust YNB-F to pH 3.5.

For intracellular pH measurements, a filter-sterilized low-fluorescence YNB-F^pH^ medium was used [0.17% YNB-F without folic acid and riboflavin, Formedium, 0.4% (NH_4_)_2_SO_4_, 2% glucose, pH level of 5.8 adjusted with NH_4_OH].

For RNA isolation, cells were grown to the mid-exponential phase either in YNB supplemented with 30 mM KCl (non-starved cells) and then starved for potassium in YNB-F medium (K^+^-starved cells) or cultivated in YNB medium with 1 M NaCl or YNB medium buffered at pH 4.5 or 7.5 with citric acid or 20 mM HEPES, respectively.

### Growth tests

Drop tests on solid media were used to compare the growth of strains. Ten-fold serial dilutions of fresh cell suspensions were spotted on YNB or low-potassium YNB-F plates. In some experiments, KCl was added to the medium as indicated in the text, or the media were supplemented with the indicated amounts of NaCl, LiCl, hygromycin B, or tetramethylammonium (TMA). The growth of cells was monitored for 2–4 days. The tests were repeated at least twice and a representative result is shown.

### Plasmids and plasmid construction

#### Construction of CRISPR plasmids

The CRISPR plasmids for generating deletions of *KmHAK1* or *KmTRK1* genes were constructed according to the protocol by Rajkumar and Morrissey (2022). Briefly, complementary oligonucleotides of the gRNA protospacer sequences predicted by the software SgRNAcas9 (Xie et al. 2014, Varela et al. 2018) were designed and synthesized with 5′ and 3′ overhangs (5′-CGTC-3′ and 5′-AAAC-3′) on the sense and antisense strands, respectively. The complementary oligonucleotides were denatured, annealed, and phosphorylated with 1 µl of T4 polynucleotide kinase (10 U/µl, NEB) in a total reaction volume of 10 µl. Fifty femtomoles of the annealed protospacer insert were then joined with 100 ng of pUCC001 in a BsaI/T7 ligase Golden Gate reaction. Bacterial transformants of reaction products were selected on ampicillin-supplemented LB medium, and colonies harbouring plasmids with correctly inserted gRNA protospacer DNA were confirmed by PCR (polymerase chain reaction) using primer Bsa-R (Table S3) and the sense oligonucleotide of each gRNA protospacer sequence.

#### Construction of gene expression plasmids

The plasmids used to express *KmHAK1* and *KmTRK1* genes in *S. cerevisiae* are listed in Table S2. All genes encoding the studied K^+^ importers were expressed from plasmids under the control of the weak and constitutive *ScNHA1* promoter. Homologous recombination was used to construct pKmHAK1 and pKmHAK1-GFP plasmids, respectively. PCR amplified *KmHAK1* with the oligonucleotides listed in Table S3 was inserted into YEp352 or pGRU1 by replacing the *ScNHA1* gene in pNHA1-985 or pNHA1-985GFP, respectively, similarly as performed previously for *ScTRK1* and *KmTRK1* (Table S2). The GFP-encoding sequence was added in-frame to the 3′ end of *KmHAK1* in pKmHAK1-GFP. *Escherichia coli* XL1-Blue was used for plasmid amplification, and successful plasmid construction was checked by restriction analysis and sequencing.

To reintegrate *HAK1* or *TRK1* genes into the genome of the *K. marxianus hak1Δtrk1Δ* strain, integrative plasmids harbouring the genes were constructed by inserting each gene into the plasmid pI4-MTU-DO-HPH (Addgene ID. 160216) by means of Golden Gate assembly (Rajkumar et al. 2019) for yeast expression under the control of the *KmGDH2* promoter (Table S2). The oligonucleotides used are listed in Table S3. The correct sequence of the genes in plasmids was verified by sequencing.

### Construction of *K. marxianus* deletants and reintegrants

Single and double mutants lacking *HAK1* and/or *TRK1* genes were constructed in *K. marxianus* NBRC1777 *lig4Δ* (KmASR.005) by an optimized lithium acetate/PEG transformation method (Rajkumar and Morrissey 2022). Briefly, cultures were transformed with CRISPR plasmids alongside repair DNA with 95 bp homology flanks for each gene. Transformants were selected on YPD supplemented with 200 mg/l hygromycin and those with successful gene deletions were identified by colony PCR. Before further work, isolated deletion strains had the CRISPR plasmids removed by passaging on liquid and solid YPD media. To reintegrate *KmHAK1* and *KmTRK1* into the genome of *hak1Δtrk1Δ*, integrative cassettes of each gene were first obtained by digestion of the respective integrative plasmids with SgsI (FD1894, Fisher Scientific). The digestion product was subsequently used to transform the strain, and transformants were selected on YPD agar supplemented with 200 mg/l hygromycin. Isolates with the cassettes correctly integrated were identified by colony PCR.

### Fluorescence microscopy

BYT12 cells producing GFP-tagged *Km*Hak1 or *Km*Trk1 were grown to OD_600_ 0.2–0.5 in YNB medium. An Olympus BX53 microscope with a Cool LED light source with 460 nm excitation and 515 nm emission and an Olympus camera DP73 were used to view the fluorescence signal. Whole-cell images were obtained with Nomarski optics.

### Relative membrane potential measurement

The relative plasma membrane potential was estimated using an assay based on the redistribution of the fluorescent dye 3,3′-dipropylthiacarbocyanine iodide (diS-C_3_(3); 0.1 mM stock solution in ethanol) (Kodedova and Sychrova 2015). Cells grown in YNB supplemented with 50 mM KCl to OD_600_ 0.2–0.5 were harvested and either directly used for membrane-potential measurements or starved of K^+^ in YNB-F medium for 2 h. Non-starved cells and cells after K^+^ starvation were washed and resuspended in 10 mM MES (pH 6.0 adjusted with triethanolamine) to OD_600_ = 0.2. The diS-C_3_(3) probe was added to the cells to a final concentration of 0.02 µM. Fluorescence emission spectra were determined every 3–5 min over 40–60 min in a Fluoromax 4 spectrofluorometer equipped with a xenon lamp. The excitation wavelength was 531 nm, and the emission range was 560–590 nm. The staining curves corresponded to the dependence of the fluorescence emission maximum wavelength λ_max_ on the staining time. In some experiments, KCl was added to the cell suspensions to a final concentration of 30 mM after 15 min of staining, as indicated in the text; the same amount of water was added to control cells. The experiments were repeated three times, and either a mean value of λ_max_ measured after 20 min of staining ± SD or a representative staining curve is shown.

### Glucose-induced acidification of the extracellular medium

Glucose-induced medium acidification was measured as described previously (Maresova and Sychrova 2007). Cells from an overnight culture grown in YNB supplemented with 100 mM KCl were washed and resuspended either in YNB or in YNB-F media without glucose and with a pH indicator (0.01% bromocresol green sodium salt) to OD_600_ = 0.1. After 120 min, medium acidification was started by adding glucose (2% final concentration); the same amount of water was added to control cells. In some experiments, KCl was added to cells in YNB-F medium to a final concentration of 30 mM together with glucose, as indicated in the text. Changes in absorbance at 595 nm were followed in an ELx808 Absorbance Microplate Reader (BioTek). The experiments were repeated three times, and the results are presented as either curves showing the changes in pH over time or means of measured extracellular pH levels ± SD.

### Intracellular pH measurement

For estimations of intracellular pH, BYT12^pHl^ cells producing pHluorin and various K^+^ importers were cultivated in YNB-F^pH^ supplemented with 100 mM KCl to OD_600_ ∼0.5. The fluorescence intensities were recorded using a Cytation 3 microplate reader (BioTek) equipped with monochromator optics (excitation wavelengths 395 nm and 475 nm, emission 508 nm) according to (Albacar et al. 2021). A culture of BYT12 that did not produce pHluorin was grown in parallel, and the corresponding background fluorescence values were subtracted from the fluorescence measured at each excitation wavelength. The ratios of emission intensity I_395nm_/I_475nm_ were used to calculate the values of intracellular pH as described previously (Zimmermannova et al. 2015). Eight technical replicates were measured for each strain within individual experiments. The measurements were repeated three times, and mean values ± SD are presented.

### Rb^+^ uptake

Rubidium is used as a potassium analogue to measure potassium uptake in yeast cells (Rodriguez-Navarro 2000). Cells were grown in a YNB medium supplemented with 30 mM KCl (non-starved cells). For potassium starvation, non-starved cells were washed with ultrapure sterile water and resuspended in YNB-F without adding potassium for 3 h (K^+^-starved cells). To characterize Rb^+^ uptake, non-starved cells or K^+^-starved cells were washed and suspended (OD_600_ ∼ 0.3) in the uptake buffer [10 mM MES supplemented with 2% glucose, 0.1 mM MgCl_2_ and adjusted to pH 5.8 with Ca(OH)_2_]. The required amounts of RbCl were added to the cell suspensions at time zero, and aliquots were taken at various times. Eight to twelve different concentrations of RbCl were used to deduce the kinetic parameters of transport. Cells were collected from liquid aliquots on Millipore filters (0.8 µm pore size) to estimate intracellular rubidium content and rapidly washed with 20 mM MgCl_2_. The cells were then treated with 0.2 M HCl and 10 mM MgCl_2_, and the extracts were analysed by atomic emission spectrophotometry (Ramos et al. 1990, Caro et al. 2019, Ruiz-Castilla et al. 2021a).

### RNA isolation and reverse transcription

Total RNA was extracted from *K. marxianus* cells using TRIsure (Bioline). Cells were grown under various conditions as indicated in the text, washed with sterile, cold water and resuspended in 1 ml of TRIsure and ~200 µl of 0.5-mm glass beads. For disruption, yeasts were vortexed 10 times for 1 min with at least 1 min intervals on ice, incubated for 5 min at 70°C, followed by another 10 periods of 1 min vortexing with cooling intervals.

Afterwards, a standard protocol for RNA isolation was followed. Isolated RNA samples were treated using DNase I (New England Biolabs) to remove contaminating DNA until no PCR amplification was observed without prior cDNA synthesis. RNA sample quality and quantification were performed spectrophotometrically (Nanodrop 2000C).

At least two RNA preparations were isolated for each set of experimental conditions. 1 µg from each RNA sample was retrotranscribed with an iScript™ cDNA Synthesis Kit (Bio-Rad) in three separate runs pooled together before PCR amplification.

### Real-time PCR

The primer oligonucleotides for *K. marxianus ACT1, TRK1*, and *HAK1* genes (Table S3) were designed with the NCBI primer-designing tool (https://www.ncbi.nlm.nih.gov/tools/primer-blast/). The quality of the primers was tested; efficiencies were close to 100%, and no primer dimers were observed. The PCR amplification was carried out in a mixture (20 µl final volume) with iTaq Universal SYBR^®^ Green Supermix (Bio-Rad), 1 µl of cDNA and 0.1 µM of the specific primers. PCR reactions were performed in at least triplicate. Real-time PCR conditions were an initial denaturation step, at 95°C for 3 min, followed by 40 PCR cycles consisting of 15 s of denaturation at 95°C and 30 s of annealing plus elongation at 60°C. Finally, melting curves were determined, and no primer dimers were detected. All experiments were repeated at least three times with three technical replicates for each sample.

### Sequence and statistical analyses

The *K. marxianus* NBRC1777 strain gene sequences were obtained from the NCBI nucleotide database (Inokuma et al. 2015). The membrane topology prediction of *Km*Trk1 was based on the most recent atomic-scale computational model of *Sc*Trk1 (Zayats *et al*. 2015). The positions and number of *Km*Hak1´s transmembrane domains were predicted by the Protter tool (Omasits et al. 2014), which was used for the visualization of topology of both K^+^ importers. The structural models of the transporters were obtained from the AlphaFold database (Jumper et al. 2021, Varadi et al. 2022).

Statistically significant differences were analysed either by an unpaired Student *t-*test using MS Office Excel or using GraphPad Prism 9 (one-way ANOVA and Dunnett’s test) for RT-PCR results. Significant differences are indicated with asterisks (*P < 0.05; **P < 0.01; ***P < 0.001).

## Results

### *Kluyveromyces marxianus* genome encodes two types of potassium-uptake transporters, and its Hak1 is more critical for growth under low-potassium conditions

**Figure 1.**
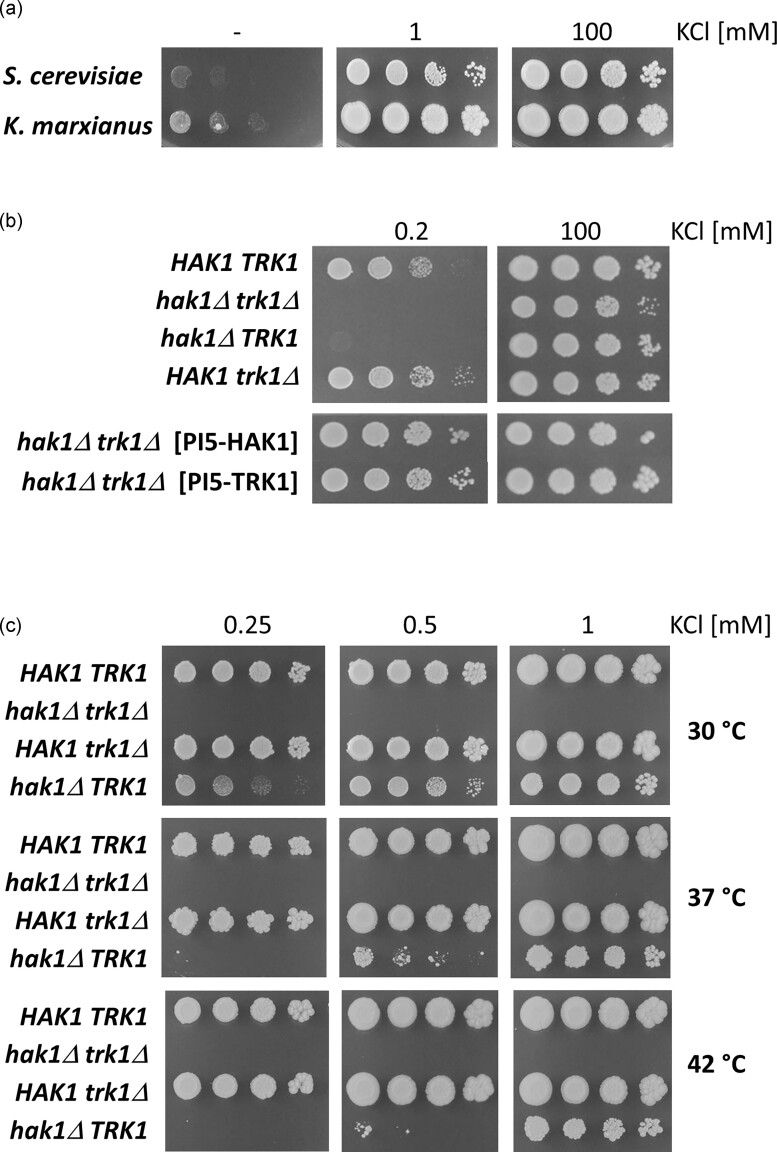
Growth of *K. marxianus* strains on low K^+^ in comparison with *S. cerevisiae* (a), in the absence of *HAK1* and/or *TRK1*, and after the reintegration of *HAK1* or *TRK1* into the double mutant (b), and at various temperatures (c). Cells were grown on YNB-F supplemented with the indicated amounts of KCl.

*Kluyveromyces marxianus* cells grow faster than *S. cerevisiae* at limiting potassium concentrations (15 µM K^+^; Fig. 1a), suggesting the presence of a powerful and high-affinity system for potassium uptake. The *K. marxianus* genome contains two ORFs for putative potassium importers: KMAR_50 362 encoding a Hak-type transporter (820 aa) and KMAR_60 040 encoding a Trk-like protein (1029 aa). We named them *KmHAK1* and *KmTRK1*, respectively. While *Km*Trk1 has already been partially characterized by its heterologous expression in *S. cerevisiae* cells (Papouskova et al. 2023), no information is available on the *Km*Hak1. To elucidate which one of the two, or whether both are involved in the observed high growth capacity of *K. marxianus* in the presence of limiting K^+^ concentrations, we constructed single and double mutants lacking one or both ORFs (cf. ‘Materials and methods’ and Table S1). We tested their growth at low and high potassium concentrations. Figure 1b shows that the deletion of *HAK1* had a dramatic negative effect on cell growth, and that *TRK1* contributed only marginally to the cell growth at 0.2 mM KCl (visible as a slight growth of the first drop of cells when comparing the double mutant and *hak1Δ TRK1* strain).

To verify that the observed growth phenotypes are linked to the loss of potassium-uptake systems, both genes were cloned into pI4-MTU-DO-HPH behind the *KmGDH2* promoter (cf. Table S2) and reintegrated back into the *hak1Δ trk1Δ* strain. The results obtained (Fig. 1b) confirmed that the reintegration of *HAK1* restored the capacity for growth at low potassium and showed that *TRK1* also supported growth well when expressed from the *GDH2* promoter. This result suggested that the expression of *TRK1* from its promoter is low when cells are grown on low potassium.

One of the most essential features of *K. marxianus* is its high thermotolerance. This species grows well above 40°C. We checked the growth phenotypes at different cultivation temperatures to determine whether the absence of Hak1 or Trk1 transporters affects thermotolerance. It was evident that it is only the activity of Trk1 which diminishes at higher temperatures (Fig. 1c, growth of *hak1Δ TRK1* mutant); the growth rate of the single mutant expressing only *TRK1* at low KCl concentrations already decreases at 37°C. On the other hand, the activity of Hak1 is robust and supports a ‘normal’ growth even at 42°C (Fig. 1c, growth of *HAK1 trk1Δ* mutant).

### The level and regulation of *KmHAK1* and *KmTRK1* expression differ

**Figure 2.**
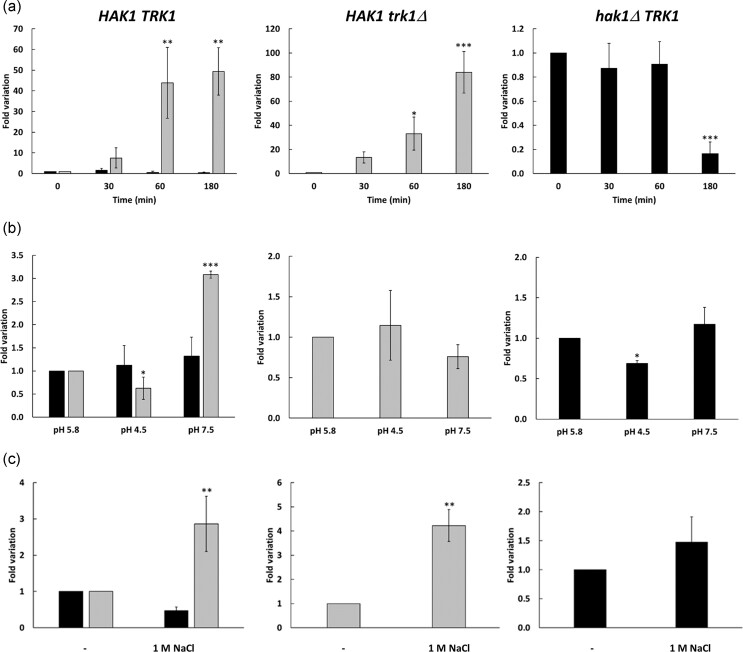
Changes in expression of *HAK1* and *TRK1* genes in *K. marxianus* strains. (a) During potassium starvation. Cells were grown to the exponential phase in YNB with 30 mM KCl (non-starved cells), washed, resuspended in YNB-F without added KCl and incubated for 3 h (K^+^-starved cells). Samples were taken at different time points, and transcript levels were compared to the beginning of starvation (time 0–expression level 1). (b) At different external pH. Cells were grown overnight to an OD_600_ of 0.6–0.7 in YNB medium buffered at pH 5.8, pH 4.5, and pH 7.5. Transcript levels were compared to pH 5.8 (level 1). (c) Upon salt stress. Cells were grown overnight in YNB medium or YNB medium, supplemented with 1 M NaCl to an OD_600_ of 0.6–0.7. Transcript levels were compared to those in cells grown without NaCl (level 1). The graphs represent the levels of *TRK1* (black) and *HAK1* (grey) transcripts. In all experiments, *ACT1* transcription was used as an internal control. Statistically significant differences are expressed as: *P < 0.05; **P < 0.01; ***P < 0.001.

To confirm that the observed difference between the growth phenotypes of cells expressing only *TRK1* (Fig. 1b strains *hak1ΔTRK1* and *hak1Δ trk1Δ* [PI5-TRK1] on 0.2 mM KCl) is based on differences in the expression level and elucidate some factors influencing the expression level of both genes, we estimated the expression in the wild type and both single mutants under a series of experimental growth conditions. First, we compared the changes in the expression of both genes upon the transfer of cells from potassium-ample (YNB + 30 mM KCl, non-starved cells) to potassium-limited conditions (YNB-F containing only 15 µM K^+^). As shown in Fig. 2a, the starvation of potassium neither changed the expression of *TRK1* in the wild type nor in the mutant lacking *HAK1* during the first 60 min of starvation. Surprisingly, prolonged starvation of the mutant lacking the *HAK1* gene significantly decreased *TRK1* expression (Fig. 2a, right panel). On the other hand, the expression of *HAK1* increased about 50 times in the wild type and almost 80 times in the *HAK1 trk1Δ* strain within 3 h of incubation in YNB-F.

When we measured the expression level of both genes at different pHs, it was evident that acidic pH did not considerably change the expression of either of the genes (Fig. 2b, pH 5.8 vs 4.5). A somewhat surprising result was obtained for pH 7.5. The expression of *HAK1* increased slightly (about three-fold) in the wild type but not in the *HAK1 trk1Δ* strain (Fig. 2b). Finally, we checked whether the expression of the two genes changed in the presence of a high concentration of NaCl. The growth of cells in the presence of 1 M NaCl resulted in a significant increase in *HAK1* mRNA levels and only minor changes in *TRK1* expression (Fig. 2c).

### Kinetic parameters of Hak1 and Trk1 differ in potassium-starved *K. marxianus* cells

To characterize the kinetic parameters of both *K. marxianus* transporters, we measured the rubidium uptake in the wild-type and mutant strains (Table 1). When the cells were grown in a sufficient amount of potassium (30 mM added to YNB medium, non-starved cells), the rubidium uptake had a similar maximum rate and half-saturation constant in all four strains, suggesting that the two transporters do not significantly contribute to Rb^+^ uptake under these conditions. The affinity of both transporters for rubidium significantly increased, as did the rate of uptake, when the cells were starved of potassium. Both transporters were in a high-affinity mode, and the K_T_ of Hak1 was about 10-fold lower than that of Trk1 (Table 1). Hak1 in the single mutant (*HAK1 trk1Δ*) also had a higher V_max_ than the Trk1 expressing mutant (*hak1Δ TRK1*), 26 vs 15 nmol mg^−1^ min^−1^, respectively, confirming the higher expression of *HAK1* during potassium starvation.

**Table 1.** Kinetic parameters of Rb^+^ transport in *K. marxianus* with a ‘normal’ potassium content and in potassium-starved cells.

	Normal K^+^ cells		K^+^-starved cells	
Strain	V_max_ (nmol mg^−1^ min^−1^)	K_T_ (mM)	V_max_ (nmol mg^−1^ min^−1^)	K_T_ (mM)
*TRK1 HAK1* (wt)	**3.82** ± 0.50	**24.60** ± 0.24	**13.26** ± 0.62	**0.06** ± 0.01
*HAK1 trk1Δ*	**3.67** ± 0.42	**19.50** ± 0.21	**26.02** ± 6.06	**0.04** ± 0.01
*hak1Δ TRK1*	**3.79** ± 0.67	**16.08** ± 0.17	**15.11** ± 3.01	**0.43** ± 0.02
*trk1Δ hak1Δ*	**4.10** ± 0.71	**18.14** ± 0.18	**5.02** ± 0.32	**14.81** ± 0.92

When we compared the kinetic parameters of Rb^+^ uptake via Trk1 and their changes upon starvation in *K. marxianus* and *S. cerevisiae* (Masaryk and Sychrova 2022), we could conclude that the maximum velocity is highly similar under both starved and non-starved conditions. Still, the affinity for Rb^+^ in non-starved cells differs. *Sc*Trk1 has 2.5 mM K_T_ (Masaryk and Sychrova 2022)_._ In contrast, the transport of Rb^+^ via *Km*Trk1 under non-starved conditions is probably negligible as the K_T_ of rubidium uptake is almost the same in cells with *TRK1* and cells without any K^+^ importer (Table 1).

### The phenotypes of *S. cerevisiae* cells expressing *KmHAK1* or *KmTRK1* differ

To compare the properties and role of *Km*Hak1 and *Km*Trk1 in more detail, we expressed them in an *S. cerevisiae* strain lacking its own Trk1 and Trk2 transporters (strain BYT12, Table S1). This approach allowed us to compare the phenotypes and properties of the resulting *S. cerevisiae* cells with those expressing other Trk and Hak transporters (previously studied by our groups) under the same expression conditions (multicopy YEp352-based plasmids behind a weak and constitutive *ScNHA1* promoter, C-terminal GFP tagging, Table S2).

**Figure 3.**
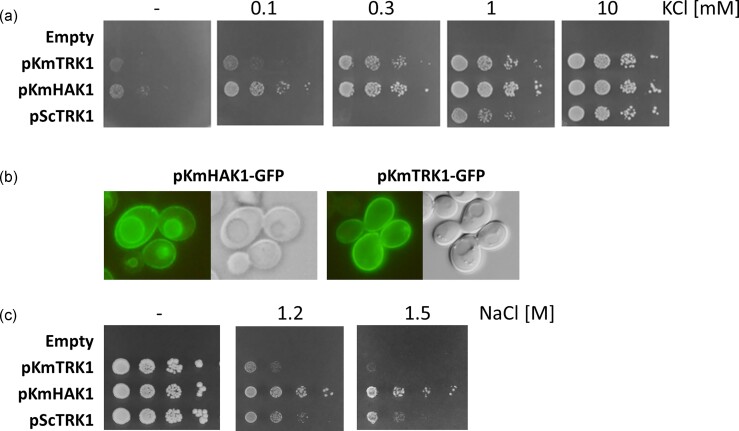
Expression of *KmHAK1* and *KmTRK1* in *S. cerevisiae. Saccharomyces cerevisiae* BYT12 cells (*trk1Δ trk2Δ*) were transformed with an empty plasmid or with pKmHAK1, pKmTRK1, pScTRK1, pKmHAK1-GFP, and pKmTRK1-GFP plasmids. (a) Growth of transformants on low K^+^. The growth of transformants was compared on YNB-F plates supplemented with the indicated amounts of KCl. (b) Localization of *Km*Hak1 and *Km*Trk1 proteins. Fluorescence (left) and Nomarski (right) pictures of BYT12 cells transformed with plasmids harbouring the genes tagged with GFP-encoding sequence at their 3′ termini. (c) Tolerance of cells to salt stress. The growth of transformants was compared on YNB plates supplemented with the indicated amounts of NaCl.

**Figure 4.**
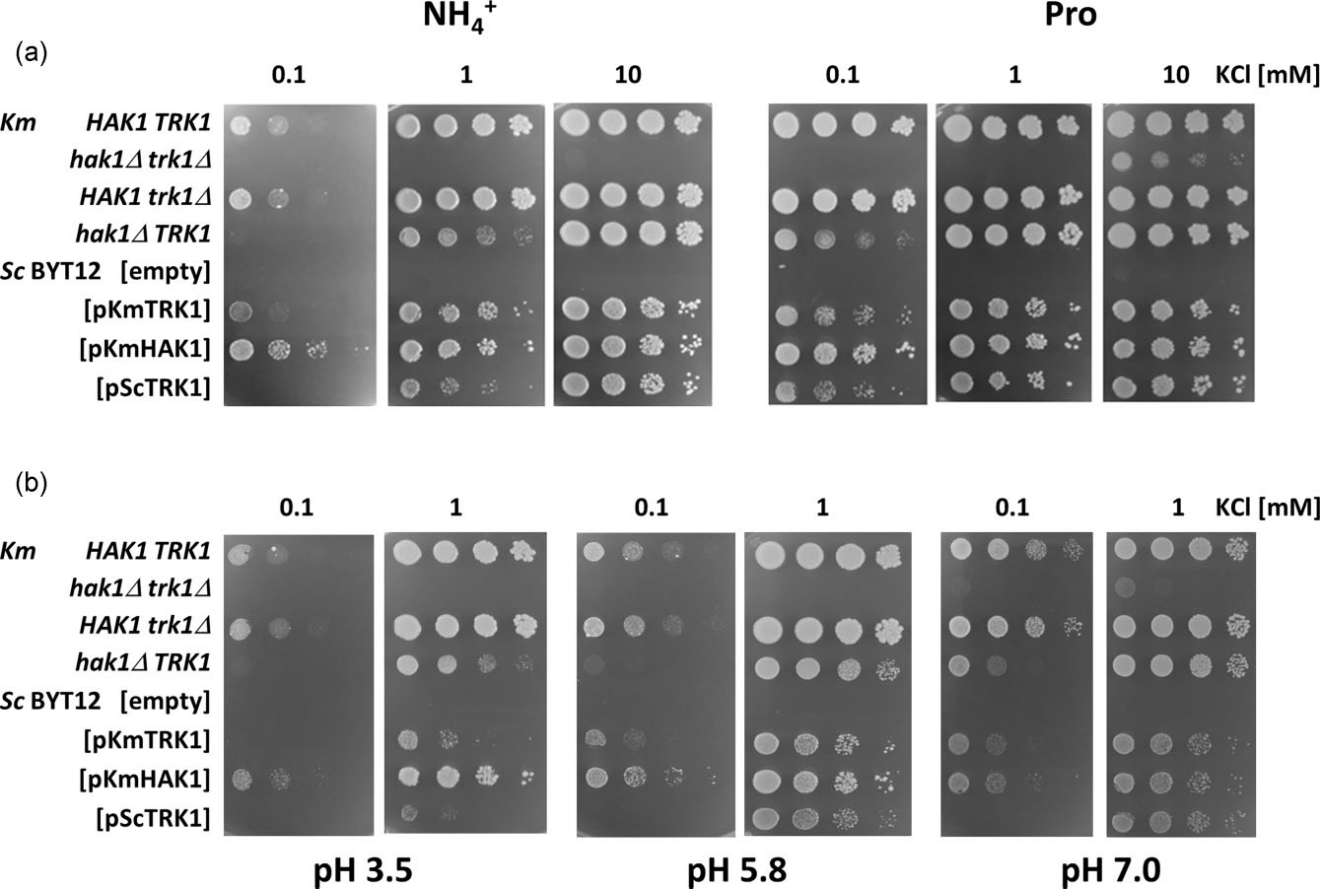
Comparison of growth of *K. marxianus* and *S. cerevisiae* strains expressing *K. marxianus* transporters. Four strains of *K. marxianus* (wild type *HAK1 TRK1*, single mutants *hak1Δ TRK1, HAK1 trk1Δ*, and double mutant *hak1Δ trk1Δ*) and four strains of *S. cerevisiae* BYT12 (harbouring empty plasmid, pKmTRK1, pKmHAK1, or pScTRK1) were grown: (a) on YNB-F plates supplemented with either 0.4% (NH_4_)_2_SO_4_ or 0.1% proline, and with the indicated amounts of KCl; (b) on YNB-F plates with different pH levels and the indicated amounts of KCl.

The first results showed that both transporters complement the inability of *S. cerevisiae* BYT12 cells to grow at low potassium concentrations, and that the presence of *Km*Hak1 improved growth better than that of *Km*Trk1. Simultaneously, *Km*Trk1 complemented the absence of chromosomal copies of *ScTRK1* and *ScTRK2* better than *Sc*Trk1 expressed from YEp352 and the *NHA1* promoter (Fig. 3a). This result is most likely a consequence of the negative impact of the presence of high ammonium concentrations on the ability of *Sc*Trk1 to support the growth of cells at low K^+^ levels (Papouskova et al. 2023); in agreement, both Trk1 proteins supported a similar rate of growth of BYT12 cells in proline-supplemented YNB-F medium (Fig. 4a).

When we visualized the C-terminally GFP-tagged versions of both transporters (Fig. 3b), it was evident that part of the *Km*Hak1 molecules did not reach the plasma membrane and seemed to be partly mislocalized to the vacuolar membrane. When we compared the growth phenotypes of BYT12 cells expressing either non-tagged or tagged *Km*Hak1 versions, it appeared that GFP-tagging diminished the capacity of cells to grow at low potassium concentrations (as shown in Fig. S1 for three independent strains for each of the two constructs). On the other hand, GFP tagging did not affect the *Km*Trk1 localization nor the growth phenotypes of cells (Fig. 3b and Fig. S1).

As was mentioned above, efficient potassium uptake via specific transporters counteracts the sensitivity of cells to high sodium concentrations. In our experiments (Fig. 3c), we could show that the presence of *Km*Hak1 in *S. cerevisiae* BYT12 cells very effectively, and much more than *Km*Trk1 or *Sc*Trk1, improved the growth of cells in the presence of high concentrations of NaCl and moderate concentrations of potassium (15 mM in YNB).

When the kinetics parameters of rubidium uptake in potassium-starved *S. cerevisiae* cells were estimated, it turned out that both heterologously expressed *K. marxianus* transporters reached a high-affinity state in *S. cerevisiae*. The half-saturation constants of all three transporters were comparable (60 and 40 µM for *Km*Trk1 and *Sc*Trk1, respectively (Masaryk and Sychrova 2022, Papouskova et al. 2023) and 29.6 ± 6 µM for *Km*Hak1).

### *Km*Hak1 supports growth more efficiently than *Km*Trk1 or *Sc*Trk1

To characterize and compare in more detail the function of *K. marxianus* transporters, we performed a series of drop tests with the four *K. marxianus* strains (wt, two single and one double mutant) and four *S. cerevisiae* BYT12–derived strains (without any potassium transporter, or with *Km*Hak1, *Km*Trk1 or *Sc*Trk1 expressed under the same conditions). The tests with low potassium concentrations (Fig. 4a) unambiguously revealed that *Km*Hak1 supported the best growth of both *K. marxianus* and *S. cerevisiae* cells. Figure 4a also shows that the presence of ammonium cations as the source of N in the growth medium diminished the ability of all eight strains to grow on plates with low potassium concentrations compared to their growth on plates with proline as a N source.

When we adjusted the growth media to different pH values (Fig. 4b), it was again the presence of *Km*Hak1 which best supported growth. It enabled both *K. marxianus* and *S. cerevisiae* to grow relatively well at acidic pH (3.5). The presence of *Km*Trk1 improved their growth slightly better than that of *Sc*Trk1 (Fig. 4b, pH 3.5, 1 mM KCl). At pH 7, the differences in growth capacity were only observed with the lowest (0.1 mM) K^+^ concentration (again, with *Km*Hak1 supporting growth the best), and they vanished with higher KCl concentrations.

An interesting difference was observed for *K. marxianus* and *S. cerevisiae* cells lacking potassium transporters (*K. marxianus hak1Δ trk1Δ* vs *S. cerevisiae* BYT12 transformed with the empty vector). Only *K. marxianus* cells lacking both potassium transporters could grow in 100 mM KCl at pH 3.5 (Fig. S2).

### *Km*Hak1 and *Km*Trk1 contribute to the tolerance of toxic cations and cationic drugs differently in *K. marxianus* and *S. cerevisiae* cells

**Figure 5.**
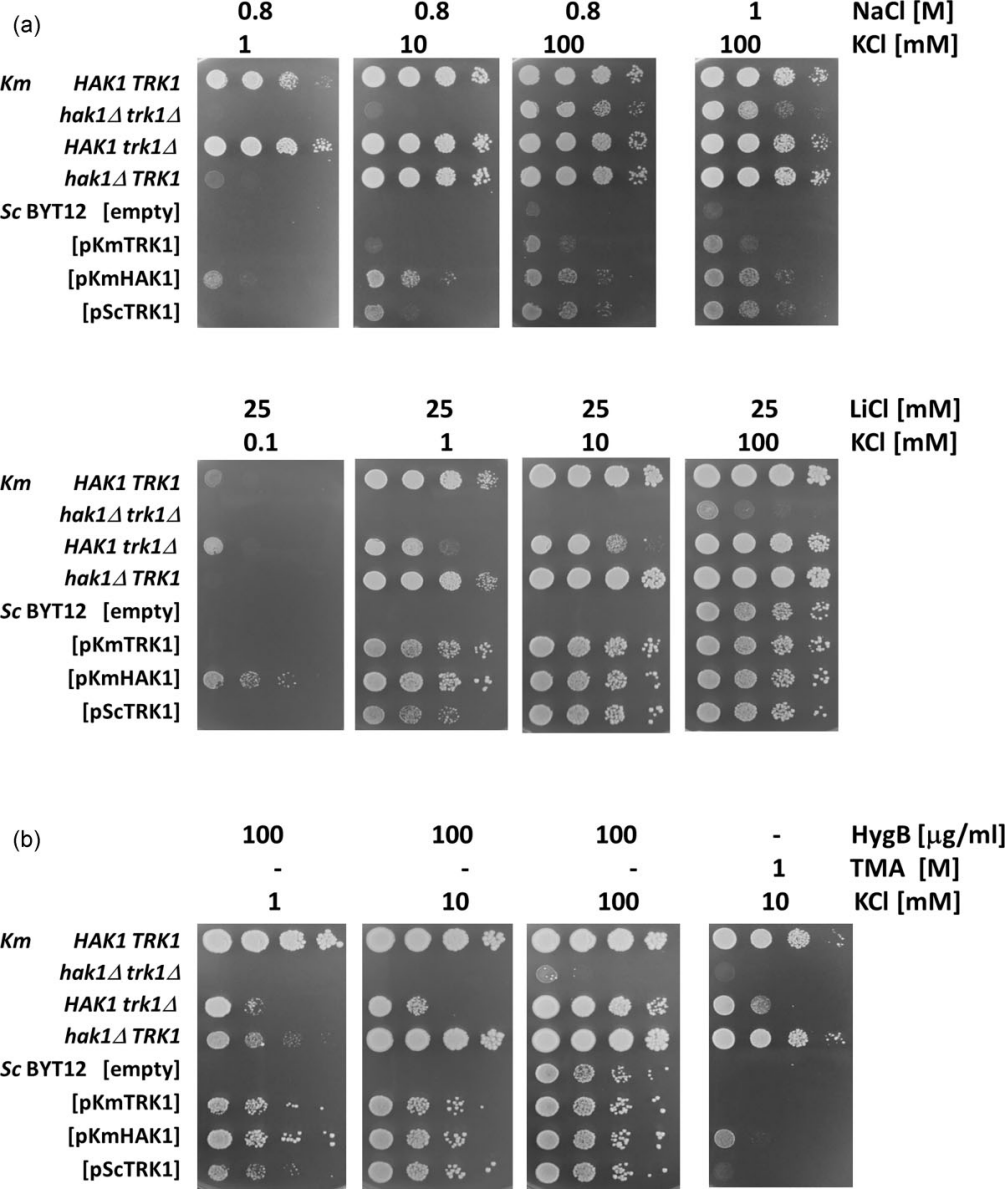
Comparison of growth of *K. marxianus* and *S. cerevisiae* strains expressing *K. marxianus* transporters in the presence of alkali metal cations and cationic drugs. Four *K. marxianus* strains (wild type *HAK1 TRK1*, single mutants *hak1Δ TRK1, HAK1 trk1Δ* and double mutant *hak1Δ trk1Δ*) and four strains of *S. cerevisiae* BYT12 (harbouring empty plasmid, pKmTRK1, pKmHAK1, or pScTRK1) were grown: on YNB-F plates supplemented with the indicated amounts of KCl and (a) NaCl or LiCl; (b) Hygromycin B or tetramethylammonium (TMA).

Based on the results shown in Fig. 3c, we decided to study the contribution of *K. marxianus* transporters to the tolerance of cells to toxic sodium and lithium cations and some harmful cationic drugs. Lithium, similarly to sodium, enters cells non-specifically and is toxic at much lower concentrations than sodium (Perkins and Gadd 1993). On the other hand, higher concentrations of NaCl, besides the toxicity of Na^+^, also cause osmotic stress to the cells. The drop tests in Fig. 5a revealed that *K. marxianus* cells tolerated higher Na^+^ concentrations if they had a functional Hak1 system. For them, a relatively low concentration of KCl (1 mM) was sufficient to support their growth when sodium was present at an 800-fold higher concentration (YNB-F with 1 mM KCl and 800 mM NaCl). Trk1 only contributed to the *K. marxianus* tolerance of high salt concentrations if higher (at least 10 mM) potassium concentrations were also present. Surprisingly, the situation was the opposite for the highly toxic lithium cations. It was the presence of Trk1 that improved *K. marxianus* growth more than the presence of Hak1 (YNB-F supplemented with 1 or 10 mM KCl and 25 mM LiCl).

This discrepancy might be due to the two experiments’ different physiological conditions (much higher osmotic pressure in the plates with NaCl). For this reason, we also tested the sensitivity of strains to the toxic cationic drugs hygromycin B and tetramethylammonium (TMA), whose influx into cells is also non-specific and driven by membrane potential. As can be seen from Fig. 5b, it was again the Trk1 system that supported the *K. marxianus* tolerance to these drugs better than Hak1 (cf. YNB-F plates with 10 mM KCl and 100 µg ml^−1^ Hyg or 1 M TMA). These results suggested that whereas Hak1 is necessary for *K. marxianus*’ survival of salt (and osmotic) stress, Trk1’s activity prevents the cell sensitivity to toxic cations. The role of a Hak transporter in improving salt tolerance was also shown for a plant HAK5 transporter expressed in yeast (Aleman et al. 2014). The effect of Trk1 (membrane-potential driven uniporter) on toxic–cation tolerance is based on its consumption of the membrane potential needed for the non-specific influx of these cations (Arino et al. 2010).

On the other hand, the presence of *Km*Hak1 was more powerful than *Km*Trk1 in supporting the growth of *S. cerevisiae* in the presence of both cations and drugs (Fig. 5a and b).

### *Km*Hak1 and *Km*Trk1 contribute similarly to the maintenance of *S. cerevisiae* membrane potential and cytosolic pH

**Figure 6.**
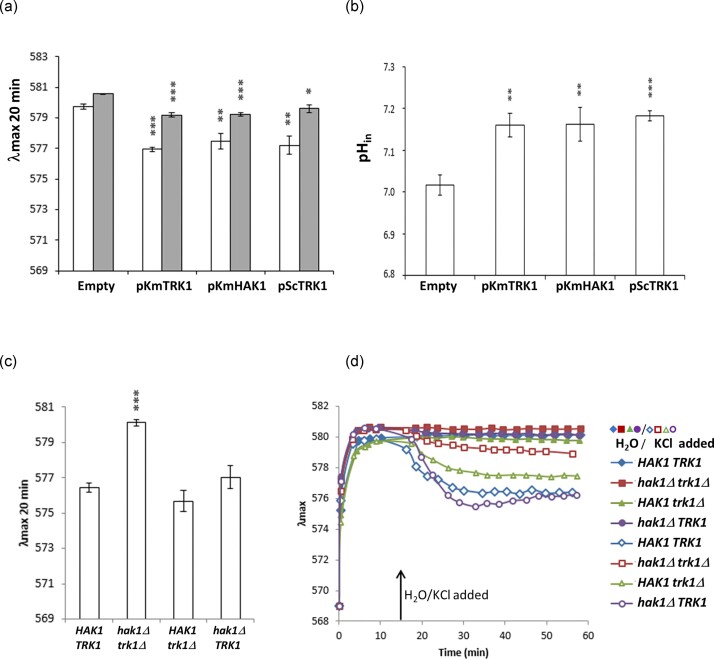
Contribution of *Km*Hak1 and *Km*Trk1 to maintaining plasma-membrane potential and intracellular pH. (a) *Saccharomyces cerevisiae* BYT12 cells (harbouring empty plasmid, pKmTRK1, pKmHAK1, or pScTRK1) were grown in YNB with 50 mM KCl (non-starved cells, white columns) and then incubated in YNB-F for 2 h (K^+^-starved cells, grey columns). Relative membrane potential was estimated using diS-C_3_(3) assay; (b) *S. cerevisiae* BYT12 ^pHl^ cells (harbouring empty plasmid, pKmTRK1, pKmHAK1, or pScTRK1) were grown in YNB-F^pH^ with 100 mM KCl, and the cytosolic pH was estimated as described in Materials and methods. (c) Four *K. marxianus* strains were grown in YNB supplemented with 50 mM KCl, and the relative membrane potential was estimated with diS-C_3_(3). (d) Four *K. marxianus* strains were grown as in (c) and subsequently starved of potassium for 2 h, then staining with the diS-C_3_(3) probe started, and KCl (empty symbols, 30 mM final concentration) or water (closed symbols) were added after 15 min. The means of three independent measurements ± SD are shown in (a)–(c). Asterisks denote statistically significant differences from the values obtained for cells with the empty plasmid (a, b) or from the wild-type strain (c); ^∗^*P* < 0.05; ^∗∗^*P* < 0.01; ^∗∗∗^*P* < 0.001. A representative result is shown in (d).

The absence of active potassium uptake in *S. cerevisiae* BYT12 cells results in the hyperpolarization of their plasma membrane, a decrease in their cytosolic pH (Navarette et al. 2010), and consequently, a lower tolerance of these cells to various positively charged ions and molecules. This hyperpolarization increases if the cells are starved of potassium (Navarette et al. 2010). The observed better tolerance of BYT12 cells expressing *K. marxianus* transporters to both alkali metal cations and cationic drugs (Fig. 5) suggested that the heterologous Trk1 and Hak1 contribute to the maintenance of *S. cerevisiae* membrane potential similarly as its own *Sc*Trk1 and *Sc*Trk2. To confirm this observation, we used a fluorescence technique (the diS-C_3_(3) assay, cf. Materials and methods) to compare the relative membrane potentials of *S. cerevisiae* BYT12 cells expressing *K. marxianus* potassium transporters. The presence of *Km*Trk1 and *Km*Hak1 in BYT12 cells was connected to significant depolarization of their relative potential under non-starved conditions, and the levels of membrane potential were similar to those of cells expressing *Sc*Trk1 (Fig. 6a). Starvation of potassium resulted in an increase in the membrane potential in all three strains expressing potassium transporters but even in the starved cells, the level of the membrane potential was lower than in control cells (BYT12 cells harbouring an empty vector).

*Saccharomyces cerevisiae* BYT12 cells have a lower cytosolic pH than cells with functional potassium transporters even if they are grown in the presence of ample potassium, i.e. under the conditions in which they have the same growth rate as cells with functional Trk1 and Trk2 (Navarette et al. 2010). To estimate and compare the values of cytosolic pH, we used pHluorin (a derivative of GFP), whose fluorescence changes according to the surrounding pH (Miesenbock et al. 1998). A sequence encoding pHluorin was integrated into the genome of BYT12 cells [cf. ‘Materials and methods’ and (Zimmermannova et al. 2015]. BYT12^pHl^ cells were transformed with plasmids (empty or encoding studied transporters), grown in 100 mM KCl and their cytosolic pH was measured. As summarized in Fig. 6b, the presence of both *Km*Hak1 and *Km*Trk1 increased the cytosolic pH, reaching values similar to those measured with cells expressing their own *Sc*Trk1. This result suggested that the activity of both *K. marxianus* transporters, a uniporter and a K^+^-H^+^ symporter, contributed to the regulation of *S. cerevisiae* Pma1 H^+^-ATPase similarly and that the activity of potassium importers is needed for proper Pma1 functioning even in the presence of surplus (100 mM) KCl.

### Both *Km*Hak1 and *Km*Trk1 contribute to the maintenance of appropriate membrane potential in *K. marxianus* non-starved cells, and *Km*Trk1 is more important for potassium-starved cells

Our results (Fig. 5a and b) were confusing concerning the involvement of *Km*Hak1 or *Km*Trk1 activity in regulating membrane potential. The results obtained with Li^+^, TMA, and Hygromycin B, together with relatively low concentrations of K^+^, suggested that *Km*Trk1 plays the primary role. Its absence probably led to a higher hyperpolarization, visible as a higher sensitivity to these three compounds (Fig. 5). Conversely, cells lacking Hak1 were more sensitive to high NaCl concentrations than cells without Trk1. To elucidate the role of both transporters in the membrane-potential levels of *K. marxianus*, we first optimized the diS-C_3_(3) assay for this yeast species. Then, we estimated the relative membrane potential of cells grown in the ample potassium (YNB + 50 mM KCl). The obtained results (Fig. 6c) revealed that both transporters are involved in maintaining membrane potential to a similar level, even when active potassium uptake is not crucial for growth (~60-70 mM K^+^ in the experimental conditions). The presence of only one of them (single mutants) is sufficient to maintain the membrane potential to the wild-type level, and only the absence of both of them leads to a significant hyperpolarization of the *K. marxianus* non-starved-cell plasma membrane.

To verify the possible different contributions of the two transporters to the maintenance of membrane potential in potassium-starved cells, cells were starved of K^+^ in YNB-F before the diS-C_3_(3) assay, and KCl was added to the starved cells 15 min after the fluorescent probe (Fig. 6d). The obtained results confirmed that the addition of KCl is reflected as a depolarization of cell membranes, that this depolarization is negligible in the double mutant lacking both transporters, and finally that Trk1’s presence is more involved in this depolarization than that of Hak1.

### Both *Km*Hak1 and *Km*Trk1 contribute to the regulation of Pma1 activity in *K. marxianus* cells

**Figure 7.**
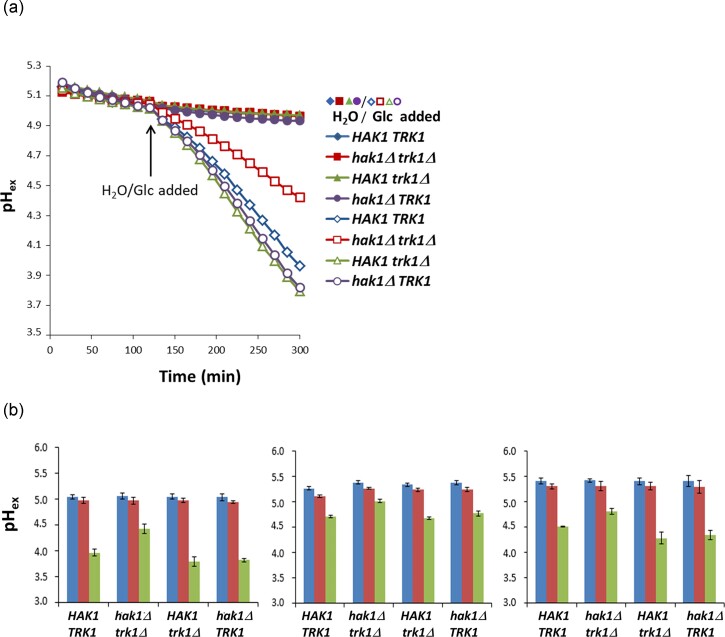
*Km*Hak1 and *Km*Trk1 contribute to the glucose-induced acidification of *K. marxianus* growth medium. (a) Four *K. marxianus* strains (wild type *HAK1 TRK1*, single mutants *hak1Δ TRK1, HAK1 trk1Δ* and double mutant *hak1Δ trk1Δ*) were grown in YNB supplemented with 100 mM KCl and then transferred to YNB without glucose. The acidification of the medium was measured as described in ‘Materials and methods’ for 5 h. After 2 h of measurement, glucose (2% final concentration, open symbols) or water (closed symbols) were added. A representative result is shown. (b) *Kluyveromyces marxianus* strains were grown as in (a) and then transferred either into YNB (left panel) or YNB-F (middle and right panels) without glucose to monitor the acidification of media. After 120 min of glucose starvation, glucose (2% final concentration) or water was added. In the right panel, 30 mM KCl was added with glucose or water. The graphs show the external pH of the media after 120 min (blue columns) and after 300 min of incubation, i.e. 180 min after the addition of water (red columns) or glucose (green columns). The means of three independent measurements ± SD are shown.

To elucidate whether the two transporters have a similar role in the regulation of Pma1 activity in *K. marxianus* cells, or whether Hak1 is more critical (cf. Fig. 4b growth of the *K. marxianus* single mutants at pH 3.5 and low K^+^), we used the approach of measuring external acidification, which is another widely used option for estimating Pma1 activity. In the first experiments, cells of the four *K. marxianus* strains were starved of glucose in YNB for 120 min. Then, glucose was added to activate the Pma1 and changes in extracellular pH were monitored for 180 min (Fig. 7a, b left panel). Before the addition of glucose, the release of protons from cells was minimal and almost the same in all four strains. The addition of glucose showed that the Pma1 in cells lacking both Hak1 and Trk1 was activated by glucose to a much lower level than in cells with at least one potassium importer. This result suggested that both transporters participate in activating *Km*Pma1 by glucose. To see whether K^+^ starvation, which especially results in *KmHAK1* upregulation (Fig. 2a), influences the ability of K^+^ importers to support Pma1 activation after the addition of glucose, we measured the external acidification with cells starved of glucose and simultaneously of potassium in YNB-F medium (Fig. 7b middle and right panels). After 120 min of glucose and K^+^ starvation, we added glucose or glucose together with KCl (final concentration 30 mM) to cells. In agreement with the necessity of active potassium uptake for Pma1 glucose-triggered activation, the acidification was lower when the measurements were done in YNB-F without added K^+^ in all strains (cf. Fig. 7b middle and right panels).

### *Km*Hak1 improves the phenotypes of *S. cerevisiae* cells with their own K^+^ transporters

**Figure 8.**
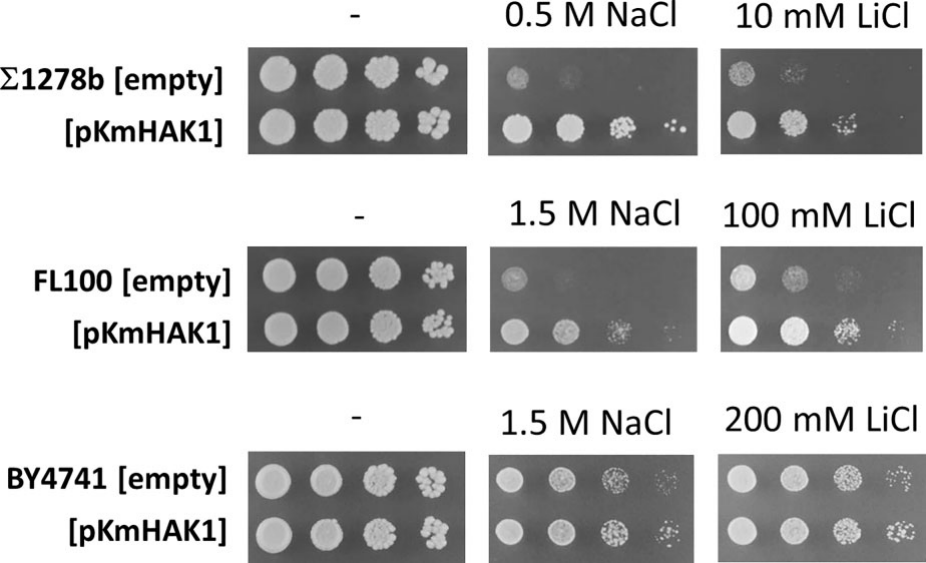
*Km*Hak1 improves the salt tolerance of *S. cerevisiae* cells. Three *S. cerevisiae* strains with functional *TRK1* and *TRK2* genes were transformed with an empty plasmid or with pKmHak1, and the growth of transformants in the presence of high concentrations of NaCl and LiCl was monitored.

The expression of some heterologous Trk transporters (e.g. *Y. lipolytica* Trk1), together with *Sc*Trk1 and *Sc*Trk2, improves the tolerance of *S. cerevisiae* cells to some types of stresses (Papouskova et al. 2023). To elucidate whether the *Km*Hak1 transporter, which turned out to be very efficient in *S. cerevisiae* BYT12 cells lacking their potassium transporters (e.g. Figs 3c, 5), could improve the growth properties and stress tolerance of *S. cerevisiae* cells with functional *Sc*Trk1 and *Sc*Trk2 transporters, we employed three different laboratory strains differing in their potassium requirements and salt-tolerance. As shown in Fig. 8, the presence of *Km*Hak1 significantly improved the tolerance to sodium and lithium of two strains (Σ1278b and FL100), and marginally that of the third one, the naturally tolerant BY4741.

## Discussion

Our analysis of the *K. marxianus* genome showed that this yeast species contains one copy of the *TRK1*, one copy of the *HAK1* genes and no *ACU1* gene, similar to many other non-conventional yeasts (Ramos et al. 2011). The two encoded transport systems belong to different protein families (Arino et al. 2019) and thereby differ in the mechanism of transport (Trk1 being a uniporter and Hak1 a K^+^–H^+^ symporter), in the protein lengths (1029 vs 820 aa residues, 20.1% identity) and also in their predicted structures (Fig. S3).

Our first results showed that the deletion of *HAK1* has a more dramatic effect on growth at low potassium concentrations than that of *TRK1* (Fig. 1b), showing that Hak1 is more important than Trk1 for the growth of *K. marxianus* under potassium-limited conditions. This drop-test result was supported by the observed strong upregulation of *HAK1* expression upon potassium starvation (Fig. 2a). Surprisingly, the simultaneous absence of both genes in the double mutant slightly affected the cell growth even at relatively high potassium concentrations (e.g. 100 mM; Fig. 1b), suggesting that in *K. marxianus*, the two transporters are partially active, in contrast to *S. cerevisiae*, in which the K^+^-specific transporters are superfluous under the same growth conditions (Navarette et al. 2010). For all the studied strains (four of *K. marxianus* and four of *S. cerevisiae*), we also observed a diminished ability to grow on plates with low potassium concentrations and ammonium sulphate compared to their growth on plates with proline as a N source (Fig. 4a). This well-known phenomenon has often been observed with *S. cerevisiae* [e.g. in (Papouskova et al. 2023)]. Ammonium cations at higher concentrations ((NH_4_)_2_SO_4_ used at 0.4% concentration in the growth media as standard) are somehow toxic to the cell growth if potassium is present at concentrations lower than 10 mM. This is probably due to a similar size of K^+^ and NH_4_^+^ cations and, consequently, the possible uptake of ammonium through the potassium-specific transporters, resulting in a competitive inhibition between the two substrates (high-affinity for K^+^ and low-affinity for NH_4_^+^).

Also, the results comparing the tolerance of cells to high NaCl concentrations revealed that the presence of *Km*Hak1 is more important than that of *Km*Trk1, both in *K. marxianus* and *S. cerevisiae* (Figs 5a and 3c, respectively). Sodium cations at higher concentrations are toxic for most yeast species. There are no specific sodium uptake systems; sodium enters the cells in a non-specific manner, and the inside-negative membrane potential drives its passage across the cell plasma membrane. Thus increased potassium uptake via high-affinity specific transporters (energized by the membrane potential/protonmotive force) diminishes the sodium influx, thereby contributing to cell sodium tolerance.

Our results suggest that the regulation of expression of the two genes in *K. marxianus* differs from those observed for the same genes in *D. hansenii* and *C. albicans*. In *K. marxianus*, the changes in external pH or addition of NaCl only provoke very small changes in the expression of both genes (Fig. 2b and c). In *D. hansenii*, both pH 4.5 and 7.5, or the presence of 1 M NaCl, resulted in a significant decrease in the expression of the *HAK1* and *TRK1* genes (Martinez et al. 2011), and starvation of potassium increased *DhHAK1* levels to a lower extent than in *K. marxianus* (30-fold vs 50-fold in 120 min, respectively). *C. albicans* has Hak1 and Trk1 transporters and an Acu1 K^+^-uptake ATPase. Acu1 is the transporter whose expression is tightly regulated. Its expression increases more than that of *CaHAK1* at pH 4.5, and starvation of potassium results in a 120-fold increase in *CaACU1* mRNA levels compared to only an 18-fold rise in *CaHAK1* expression (Ruiz-Castilla et al. 2021b). The response to 1 M NaCl in *C. albicans* is similar to that of *D. hansenii*; all three transporters exhibit a significant decrease in expression.

Both *K. marxianus* transporters were able to reach a high-affinity state during potassium starvation; nevertheless, the K_T_ of Hak1 was about 10-fold lower than that of Trk1 (Table 1). Hak1 in the single mutant (*HAK1 trk1Δ*) also had a higher V_max_ than the Trk1 expressing mutant (*hak1Δ TRK1*), 26 vs 15 nmol mg^−1^ min^−1^, respectively, confirming the higher expression of *HAK1* during potassium starvation. As far as we know, this is the first estimation of Hak1’s kinetic parameters and evidence of its ability to reach a very high-affinity state in potassium-starved native species. So far, the kinetic parameters for various yeast Hak1s were only estimated for heterologously expressed (in *S. cerevisiae*) transporters. When the *K. marxianus* transporters were expressed in *S. cerevisiae* cells, and their kinetic parameters of rubidium uptake were compared to those of *Sc*Trk1, the half-saturation constants of all three transporters were comparable (60 and 40 µM for *Km*Trk1 and *Sc*Trk1, respectively (Masaryk and Sychrova 2022, Papouskova et al. 2023) and 29.6 ± 6 µM for *Km*Hak1), exhibiting a significantly lower K_T_ of *Km*Trk1 in *S. cerevisiae* than in *K. marxianus* (60 and 430 µM, respectively). The differences in K_T_ of the same transporter expressed in two different species might be, among other things (e.g. conditions of expression), due to a different composition of the plasma membranes of the two yeast species. The lipid composition contributes to the membrane potential and the activity of various transporters, as shown previously for *S. cerevisiae* Trk1 (Masaryk and Sychrova 2022) or drug-efflux systems (Kodedova and Sychrova 2015).

The presence of *Km*Hak1-GFP in the vacuolar membrane (Fig. 3b) was surprising, as we did not observe it for *Ca*Hak1 expressed in the same strain under the same conditions (Elicharova et al. 2016) *Ca*Hak1 was not correctly localized to the plasma membrane either; it was most likely stacked along the secretory pathway, and its contribution to BYT12 growth in low potassium was relatively small, only improving the cell growth with 5 mM and higher KCl concentrations in the media (Elicharova et al. 2016). Thus *Km*Hak1 is better tailored for functioning in *S. cerevisiae* cells than *Ca*Hak1. This is probably not due to a relatively longer distance between *S. cerevisiae* and *C. albicans* genomes, as *Ca*Trk1 localizes very well to the *S. cerevisiae* plasma membrane and efficiently supports the growth of BYT12 under low-K^+^ conditions (Elicharova et al. 2016). In summary, *Km*Hak1 appeared to be an excellent importer of potassium in *S. cerevisiae* cells, and the possibility that proportion of its molecules are localized (upon GFP-tagging?) in the vacuolar membrane (in a functional state?) should be studied in the future. Based on our actual results, we cannot say whether the GFP-tagging causes a partial inactivation of *Km*Hak1 or partial mislocalization to the vacuole, which is reflected as a lower capacity of cells for potassium uptake at the plasma membrane.

*Saccharomyces cerevisiae* cells lacking their two K^+^ importers are highly sensitive to low external pH ((Navarette et al. 2010); Fig. S2). In contrast, the *K. marxianus* cells lacking both potassium transporters could grow in 100 mM KCl at pH 3.5 (Fig. S2), suggesting that the tolerance of *K. marxianus* to low pH is not connected as much to the active potassium importers as in *S. cerevisiae*. In this respect and in terms of the mechanism of transport (symport of K^+^ and H^+^), it was surprising that *Km*Hak1 supported growth better than Trk1 proteins (uniport of just potassium) at low external pH (Fig. 4b). At low pH, the cells need to cope with a higher inward gradient of protons across the plasma membrane. In addition, the activity of a heterologous symporter brings in many more protons in and makes it more difficult for Pma1 to maintain an appropriate cytosolic pH.

Based on monitoring the external acidification, we could conclude that both Hak1 and Trk1 transporters contribute to the regulation of *K. marxianus* Pma1 activity similarly, and the activity of *Km*Pma1 is higher when a higher concentration of K^+^ is present. Only one of the two K^+^ importers is sufficient to reach the level of acidification observed for the wild-type *K. marxianus* cells.

Although the presence of the *Km*Hak1 transporter was more advantageous for cells than the presence of *Km*Trk1 in many studied conditions, our results showed that Trk1’s presence is more important for the regulation of plasma-membrane potential. Trk1’s activity results in a deeper depolarization than that of Hak1 (Fig. 6d), in spite of the fact that K^+^ starvation results in a significant upregulation of the *HAK1* gene and not of the *TRK1* gene (Fig. 2a). The comparison of depolarization confirmed the observation from the drop tests (Fig. 5a, Li^+^ and Fig. 5b), i.e. a more prominent role of Trk1 in membrane-potential consumption and thereby in cationic-drug tolerance. In this way, its role resembles that of *S. cerevisiae* Trk2, which does not provide potassium for cell growth as much as *Sc*Trk1, but helps maintain an appropriate membrane-potential level and survive adverse conditions (Arino et al. 2010). Nevertheless, *Km*Trk1 can also act as an efficient potassium importer (e.g. Fig. 1; *hak1Δ trk1Δ* [PI5-TRK1]). When *Km*Trk1 is expressed in *S. cerevisiae* BYT12 under the same conditions as *Sc*Trk1 (the same plasmid and promoter), it provides growing cells with potassium better than *Sc*Trk1 (Fig. 3a) and contributes a little bit more to hygromycin B or lithium tolerance (Fig. 5; 1 mM KCl + 100 µg/mL HygB or 1 mM KCl + 25 mM LiCl).

## Conclusions

Our study showed that the two different potassium transporters of *K. marxianus*, Hak1 and Trk1, have only partly overlapping roles in cell physiology. *Kluyveromyces marxianus* has an enormous capacity to obtain high amounts of potassium, indispensable for cell growth, from an environment with scarce concentrations of this cation. This is due to the presence of an exceptionally effective member of the HAK family of potassium-proton symporters, *Km*Hak1. This transporter fulfils the role of the primary potassium importer; its expression is strongly upregulated, and its affinity for the substrate is very high. Its remarkable activity is visible both in *K. marxianus* and when expressed from a non-regulable promoter in *S. cerevisiae*, which lacks its own potassium uptake systems. The *KmHAK1* gene could be employed, upon integration into the genome, to improve the salt tolerance of strains used in some biotechnology processes in which the cells are grown in environments containing high concentrations of monovalent cations.

The other transporter, *Km*Trk1, seems to be more involved in other aspects of potassium homeostasis than in the acquisition of potassium for cell growth. In *K. marxianus*, its activity helps to maintain an appropriate membrane potential, consequently contributing to the tolerance of cells to highly toxic cations (e.g. Li^+^) and cationic drugs.

The only aspect in which *Km*Hak1 and *Km*Trk1 seem to contribute similarly (and in both yeast species) is the activation of plasma-membrane H^+^-ATPase Pma1 and, thereby, cytosolic pH homeostasis.

Our study also revealed that although heterologous expression in *S. cerevisiae* is an efficient tool for characterizing the basic properties of transporters (e.g. substrate specificity and affinity), their contribution to cell physiology may differ in the native species and *S. cerevisiae*. This was visible, for instance, in the differences in their contribution to the tolerance of toxic cations (differences between the two transporters in *K. marxianus* but almost the same contribution in *S. cerevisiae*) and in the different impact of their activity on the level of membrane potential in *K. marxianus*, but similar effects in their *S. cerevisiae* host.

## Supplementary Material

foae031_Supplemental_Files

## Data Availability

The data underlying this article are available in the article and in its online supplementary material.
