# Supplementary material for: The superior growth of Kluyveromyces marxianus at very low potassium concentrations is enabled by the high-affinity potassium transporter Hak1
Source: FEMS Yeast Res. 2024 Oct 3;24:foae031. doi: 10.1093/femsyr/foae031 (PMC11484806; doi:10.1093/femsyr/foae031)
Supplement: foae031_Supplemental_Files [file foae031_supplemental_files.zip › Supplementary figures legends.docx]

**Fig. S1 GFP-tagging diminishes the activity of *Km*Hak1 at the plasma membrane of *S. cerevisiae* BYT 12 cells.** The growth of three independent transformants for each of the used plasmids (pKmHak1, pKmHAK1-GFP, pKmTRK1 and pKmTRK1-GFP) at low and high KCl concentrations, respectively, was monitored and compared with the growth of transformants harbouring the empty plasmid.

**Fig. S2 Comparison of growth of *K. marxianus* and *S. cerevisiae* strains expressing *K. marxianus* transporters on YNB-F plates with different pH levels and 100 mM KCl.**

**Fig. S3 Structure of *Km*Hak1 and *Km*Trk1. (A)** Membrane topology of the *K. marxianus* K^+^ importers. **(B)** Predicted structures of *K. marxianus* K^+^ importers. The colours in the AlphaFold models correspond to their per-residue confidence scores (pLDDT values): dark blue – very high (pLDDT > 90), light blue – confident (90 > pLDDT > 70), yellow – low (70 > pLDDT > 50), orange – very low (pLDDT < 50).
