# Supplementary material for: The superior growth of Kluyveromyces marxianus at very low potassium concentrations is enabled by the high-affinity potassium transporter Hak1
Source: FEMS Yeast Res. 2024 Oct 3;24:foae031. doi: 10.1093/femsyr/foae031 (PMC11484806; doi:10.1093/femsyr/foae031)
Supplement: foae031_Supplemental_Files [file foae031_supplemental_files.zip › Table S1.docx]

**Table S1. Strains used in this study**

| Organism | Strain | Genotype | Reference |
| --- | --- | --- | --- |
| *K. marxianus* | NBRC 1777 | *lig4Δ HAK1 TRK1* | (Inokuma *et al.*, 2015) |
|  |  | *lig4Δ HAK1 trk1Δ* | This work |
|  |  | *lig4Δ hak1Δ TRK1* | This work |
|  |  | *lig4Δ trk1Δ hak1Δ* | This work |
| *S. cerevisiae* | BY4741 | *MAT***a** *his3Δ1 leu2Δ0 met15Δ0 ura3Δ0* | EUROSCARF |
|  | BYT12 | BY4741 *trk1Δ::loxP trk2Δ::loxP* | (Petrezselyova *et al.*, 2010) |
|  | BYT12^pHl^ | BYT12 *his3Δ1::loxP-kanMX-loxP- GPD1^P^-pHluorin* | (Zimmermannova *et al.*, 2015) |
|  | Σ1278b | *MAT***a** *can1 lyp1 ura3Δ* | H. Sychrova, unpublished results |
|  | FL100 | *MAT***a** *ura3Δ trp1–4* | (Kinclova *et al.*, 2001) |

Inokuma K, Ishii J, Hara KY, Mochizuki M, Hasunuma T, Kondo A 2015. Complete genome sequence of *Kluyveromyces marxianus* NBRC1777, a nonconventional thermotolerant yeast. *Genome Announc***3**.

Kinclova O, Potier S, Sychrova H 2001. The *Zygosaccharomyces rouxii* strain CBS732 contains only one copy of the *HOG1* and the *SOD2* genes. *J Biotechnol* **88:** 151-158.

Petrezselyova S, Zahradka J, Sychrova H 2010. *Saccharomyces cerevisiae* BY4741 and W303-1A laboratory strains differ in salt tolerance. *Fung Biol* **114:** 144-150.

Zimmermannova O, Salazar A, Sychrova H, Ramos J 2015. *Zygosaccharomyces rouxii* Trk1 is an efficient potassium transporter providing yeast cells with high lithium tolerance. *FEMS Yeast Res* **15:** fov029.
