# Supplementary material for: The superior growth of Kluyveromyces marxianus at very low potassium concentrations is enabled by the high-affinity potassium transporter Hak1
Source: FEMS Yeast Res. 2024 Oct 3;24:foae031. doi: 10.1093/femsyr/foae031 (PMC11484806; doi:10.1093/femsyr/foae031)
Supplement: foae031_Supplemental_Files [file foae031_supplemental_files.zip › Table S2.docx]

**Table S2. Plasmids used in this study**

| **Plasmid** | **Description** | **Source/reference** |
| --- | --- | --- |
| *Expression in S. cerevisiae*  YEp352 | Multi-copy empty vector | (Hill *et al.*, 1986) |
| pNHA1-985 | *S. cerevisiae NHA1* in YEp352 | (Kinclova *et al.*, 2001) |
| pScTRK1 | *S. cerevisiae TRK1* in YEp352 | (Papouskova *et al.*, 2023) |
| pKmTRK1 | *K. marxianus TRK1* in YEp352 | (Papouskova *et al.*, 2023) |
| pKmHAK1 | *K. marxianus HAK1* in YEp352 | This work |
| pGRU1 | Multi-copy empty with GFP | B.Daignan-Fornier, NCBI AJ249649 |
| pNHA1-985GFP | *S. cerevisiae NHA1* in pGRU1 | (Kinclova *et al.*, 2001) |
| pScTRK1-GFP | *S. cerevisiae TRK1* in pGRU1 | (Zimmermannova *et al.*, 2019) |
| pKmTRK1-GFP | *K. marxianus TRK1* in pGRU1 | (Papouskova *et al.*, 2023) |
| pKmHAK1-GFP | *K. marxianus HAK1* in pGRU1 | This work |
| *Expression in K. marxianus*  pI4-MTU-DO-HPH | Integrative, empty, Hyg^R^ | (Rajkumar and Morrissey, 2020), Addgene 160216 |
| PI5-HAK1 | *KmHAK1* behind *KmGDH2* promoter  in pI4-MTU-DO-HPH | This work |
| PI5-TRK1 | *KmTRK1* behind *KmGDH2* promoter  in pI4-MTU-DO-HPH | This work |

Hill JE, Myers AM, Koerner TJ, Tzagoloff A 1986. Yeast/*E. coli* shuttle vectors with multiple unique restriction sites. *Yeast* **2:** 163-167.

Kinclova O, Ramos J, Potier S, Sychrova H 2001. Functional study of the *Saccharomyces cerevisiae* Nha1p C-terminus. *Mol Microbiol* **40:** 656-668.

Papouskova K, Gomez M, Kodedova M, Ramos J, Zimmermannova O, Sychrova H 2023. Heterologous expression reveals unique properties of Trk K^+^ importers from nonconventional biotechnologically relevant yeast species together with their potential to support *Saccharomyces cerevisiae* growth. *Yeast* **40:** 68-83.

Rajkumar AS, Morrissey JP 2020. Rational engineering of *Kluyveromyces marxianus* to create a chassis for the production of aromatic products. *Microb Cell Fact* **19:** 207.

Zimmermannova O, Felcmanova K, Rosas-Santiago P, Papouskova K, Pantoja O, Sychrova H 2019. Erv14 cargo receptor participates in regulation of plasma-membrane potential, intracellular pH and potassium homeostasis via its interaction with K^+^-specific transporters Trk1 and Tok1. *BBA-Mol Cell Res* **1866:** 1376-1388.
