# Supplementary material for: The superior growth of Kluyveromyces marxianus at very low potassium concentrations is enabled by the high-affinity potassium transporter Hak1
Source: FEMS Yeast Res. 2024 Oct 3;24:foae031. doi: 10.1093/femsyr/foae031 (PMC11484806; doi:10.1093/femsyr/foae031)
Supplement: foae031_Supplemental_Files [file foae031_supplemental_files.zip › Table S3.docx]

**Table S3.** Oligonucleotides used in this study

| **Oligonucleotide** | **Sequence (5' → 3')** |
| --- | --- |
|  |  |
| ***K. marxianus***  Bsa-R | TACACGCGTTTGTACAGAAAAAAAAGAAAAATTTGA |
| *For deletion* |  |
| TRK1_chk_F | TTACAGGAAGACGTTAGG |
| TRK1_chk_R | ACAGAGGTATCTAAATCC |
| TRK1_RF_F | GCCCACAAACTTCATCCTATTGCAAGTTTAATATGCCCGAACTTTATTGCATTTCACTATTTTTATATTATAGGGTTTTCGATATTAGGCAGTATTAAGAAGAGATTCAT |
| TRK1_RF_R | AATCAGTAAAAGCCACCATGTTGCTGCCTTTGGGAAAAGCATAGTGAAACAACGCCGTGGATGATCCAATAAAAATCCCAATGAATCTCTTCTTAATACTGCCTAATATC |
| TRK1_gRNA_1_F | CGTCTGAGATGGTGAATAGAGACG |
| TRK1_gRNA_1_R | AAACCGTCTCTATTCACCATCTCA |
| TRK1_gRNA_2_F | CGTCTGTGAAAAGTCCTGCAGAAA |
| TRK1_gRNA_2_R | AAACTTTCTGCAGGACTTTTCACA |
| TRK1_gRNA_3_F | CGTCAGATGGGCATGATGCTGATA |
| TRK1_gRNA_3_R | AAACTATCAGCATCATGCCCATCT |
| TRK1_gRNA_4_F | CGTCCTGATGGTATCAATCCGACT |
| TRK1_gRNA_4_R | AAACAGTCGGATTGATACCATCAG |
| HAK1_chk_F | TTCTCTCGTGATAGAAGG |
| HAK1_chk_R | GTCATCATCATCATCACC |
| HAK1_RF_F | TTGCTAATATAAGTACAAGCATAGTATCAGCAGTATCGACGCCCAAAACAGACTTTTTTATTACCTATTAGCAGCAACCCTTGGCCTAAATGAGCATGTCGGTATTGCTG |
| HAK1_RF_R | CCAAGCACCATGTGGCACCTTGAGCAAGTTAGCACCGATCAAACAGCACTCGAGGGAACCGAACACGCACAAGAAGAGCACAGCAATACCGACATGCTCATTTAGGCCAA |
| HAK1_gRNA_1_F | CGTCTGCCACGTCGCCATTGTATA |
| HAK1_gRNA_1_R | AAACTATACAATGGCGACGTGGCA |
| HAK1_gRNA_2_F | CGTCAAGAGCTTCAGAAAGTGGGT |
| HAK1_gRNA_2_R | AAACACCCACTTTCTGAAGCTCTT |
| HAK1_gRNA_3_F | CGTCATTGTCCTATTGGTTATCGG |
| HAK1_gRNA_3_R | AAACCCGATAACCAATAGGACAAT |
| HAK1_gRNA_4_F | CGTCAGAGCGGTCAAGGATTCTAC |
| HAK1_gRNA_4_R | AAACGTAGAATCCTTGACCGCTCT |
| *For expression* |  |
| KmTRK1_for | GCATCGTCTCATCGGTCTCATATGTTCTTGCGTAAGCTGGTAC |
| KmTRK1_rev | ATGCCGTCTCAGGTCTCAGGATTCATGTTGTTCCTATTATAGGATC |
| KmTRK1_seq1 | AGCAGAAAGAGACTCTCGC |
| KmTRK1_seq2 | GATTTTGCAGAACATGGCTC |
| KmTRK1_seq3 | GGTCCTCTTCAAGATAAGTC |
| KmTRK1_seq4 | TCGTCAGTGCGTATGGTACC |
| KmHAK1_for | GCATCGTCTCATCGGTCTCATATGAGCACACGTGAAGGTG |
| KmHAK1_rev | ATGCCGTCTCAGGTCTCAGGATTTAGATGACGACGGTTTTAC |
| KmHAK1_seq1 | GCATGAGAGCTCTTGGCTTC |
| KmHAK1_seq2 | AGAGTATCCCAGGTAAGAGC |
| KmHAK1_seq3 | ATACCATGAAGGCTAACC |
| KmHAK1_seq4 | TGTACACATCTGTAACGAGC |
| pGDH2_seq | ACACAATAAACACTCCCC |
| *For RT-PCR* |  |
| TRK1-F | ACCAGCCAAGACTGAACCG |
| TRK1-R | GCGAGGCTTCATTTTGTCCC |
| HAK1-F | TTTTGGCTATTCACGATCACCG |
| HAK1-R | TACAACGCAATTTGACCACCC |
| ACT1-F | GGTTCTGGTATGTGTAAAGCCG |
| ACT 1-R | GTCTACCGACGATGGATGGG |
|  |  |
| ***S. cerevisiae*** |  |
| *For cloning* |  |
| KmHAK1-N | TGTACATTATAAAAAAAAATCCTGAACTTAGCTAGATATTATGAGCACACGTGAAGGTGA |
| KmHAK1-C-YEp | CACGACGTTGTAAAACGACGGCCAGTGCCAAGCTTGCATGTTAGATGACGACGGTTTTACC |
| KmHAK1-C-pGRU | TAAAGCTCCGGAGCTTGCATGCCTGCAGGTCGACTCTAGAGATGACGACGGTTTTACCGAAG |
| *For sequencing* |  |
| KmHAK-S1 | ACGGCAAGACCATGCCTACC |
| KmHAK-S2 | CGGTTTGACCGTCTCCATGG |
| KmHAK-S3 | TGTACACATCTGTAACGAGC |
| KmHAK-S4 | TTGCTACAGCGTCTTCATCG |
| KmHAK-S5 | TGCTTGATCAAGTATGCACC |
