# Supplementary figures and images for: The superior growth of Kluyveromyces marxianus at very low potassium concentrations is enabled by the high-affinity potassium transporter Hak1
Source: FEMS Yeast Res. 2024 Oct 3;24:foae031. doi: 10.1093/femsyr/foae031 (PMC11484806; doi:10.1093/femsyr/foae031)

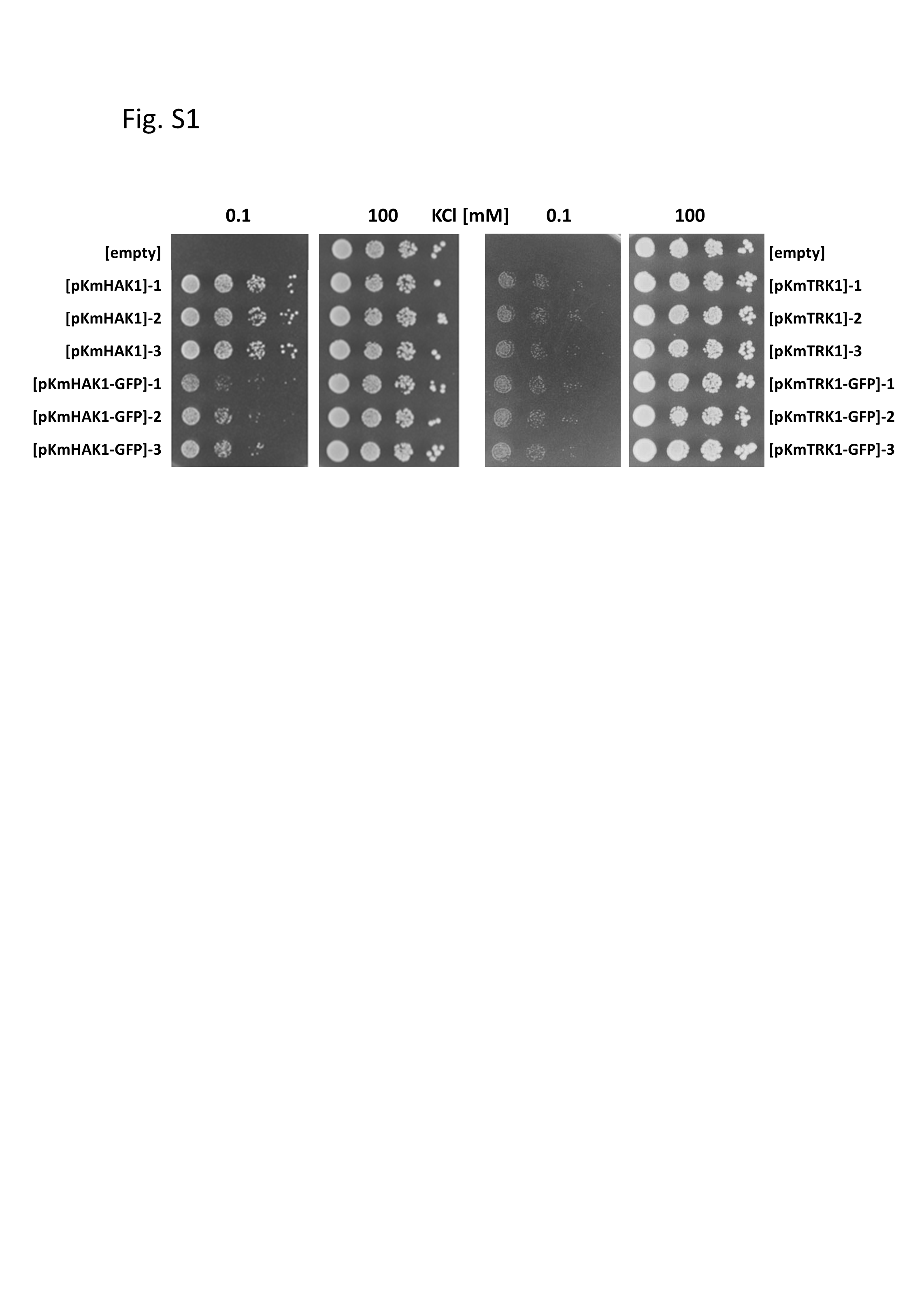

Supplement: foae031_Supplemental_Files [file foae031_supplemental_files.zip › Figure S1.TIF]

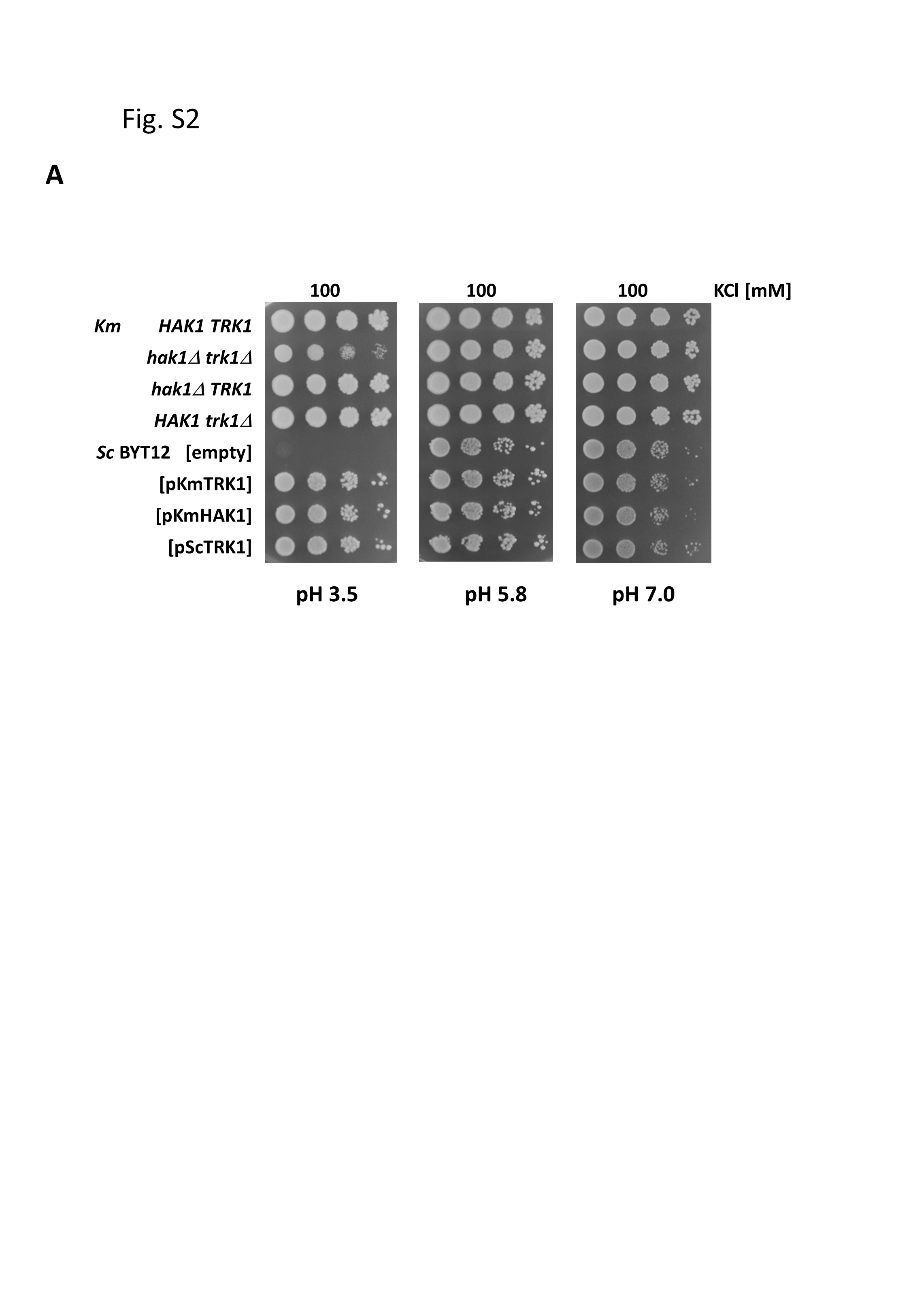

Supplement: foae031_Supplemental_Files [file foae031_supplemental_files.zip › Figure S2.TIF]

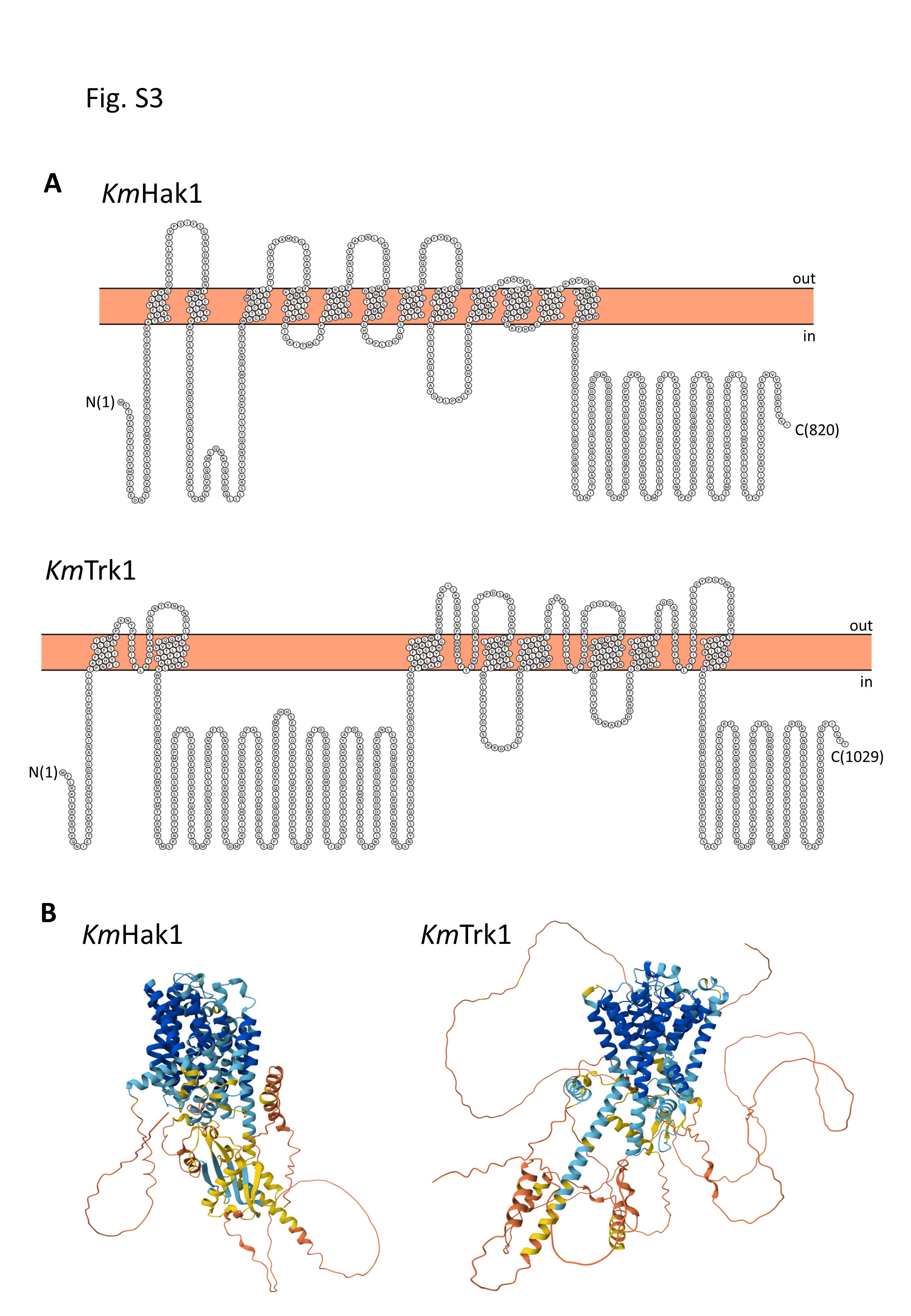

Supplement: foae031_Supplemental_Files [file foae031_supplemental_files.zip › Figure S3.TIF]
